# Determination of *Salmonella enterica* Leaf Internalization Varies Substantially According to the Method and Conditions Used to Assess Bacterial Localization

**DOI:** 10.3389/fmicb.2021.622068

**Published:** 2021-10-04

**Authors:** Madhvi Chahar, Yulia Kroupitski, Rachel Gollop, Eduard Belausov, Maeli Melotto, Shlomo Sela-Saldinger

**Affiliations:** ^1^Department of Food Sciences, The Volcani Center, Institute for Postharvest and Food Sciences, Agriculture Research Organization, Rishon-LeZion, Israel; ^2^Microscopy Unit, Plant Sciences, Ornamental Plants and Agricultural Biotechnology, The Volcani Center, Agriculture Research Organization, Rishon-LeZion, Israel; ^3^Department of Plant Sciences, University of California, Davis, Davis, CA, United States

**Keywords:** tomato, lettuce, *Arabidopsis*, internalization, attachment, colonization, disinfection, fresh produce

## Abstract

In a previous study, comparing the internalization of *S. enterica* serovar Typhimurium in various leaves by confocal microscopy, we have demonstrated that the pathogen failed to internalize tomato leaves. Numerous reasons may account for these findings, yet one such factor might be the methodology employed to quantify leaf internalization. To this end, we have systematically studied leaf localization of a Green-fluorescent protein-labeled *Salmonella* strain in tomato, lettuce, and *Arabidopsis* leaves by surface sterilization and enumeration of the surviving bacteria, side by side, with confocal microscopy observations. Leaf sterilization was performed using either sodium hypochlorite, silver nitrate, or ethanol for 1 to 7min. The level of internalization varied according to the type of disinfectant used for surface sterilization and the treatment time. Treatment of tomato leaves with 70% ethanol for up to 7min suggested possible internalization of *Salmonella*, while confocal microscopy showed no internalization. In the case of in lettuce and *Arabidopsis* leaves, both the plate-count technique and confocal microscopy demonstrated considerable *Salmonella* internalization thought different sterilization conditions resulted in variations in the internalization levels. Our findings highlighted the dependency of the internalization results on the specific disinfection protocol used to determine bacterial localization. The results underscore the importance of confocal microscopy in validating a particular surface sterilization protocol whenever a new pair of bacterial strain and plant cultivar is studied.

**GRAPHICAL ABSTRACT:**
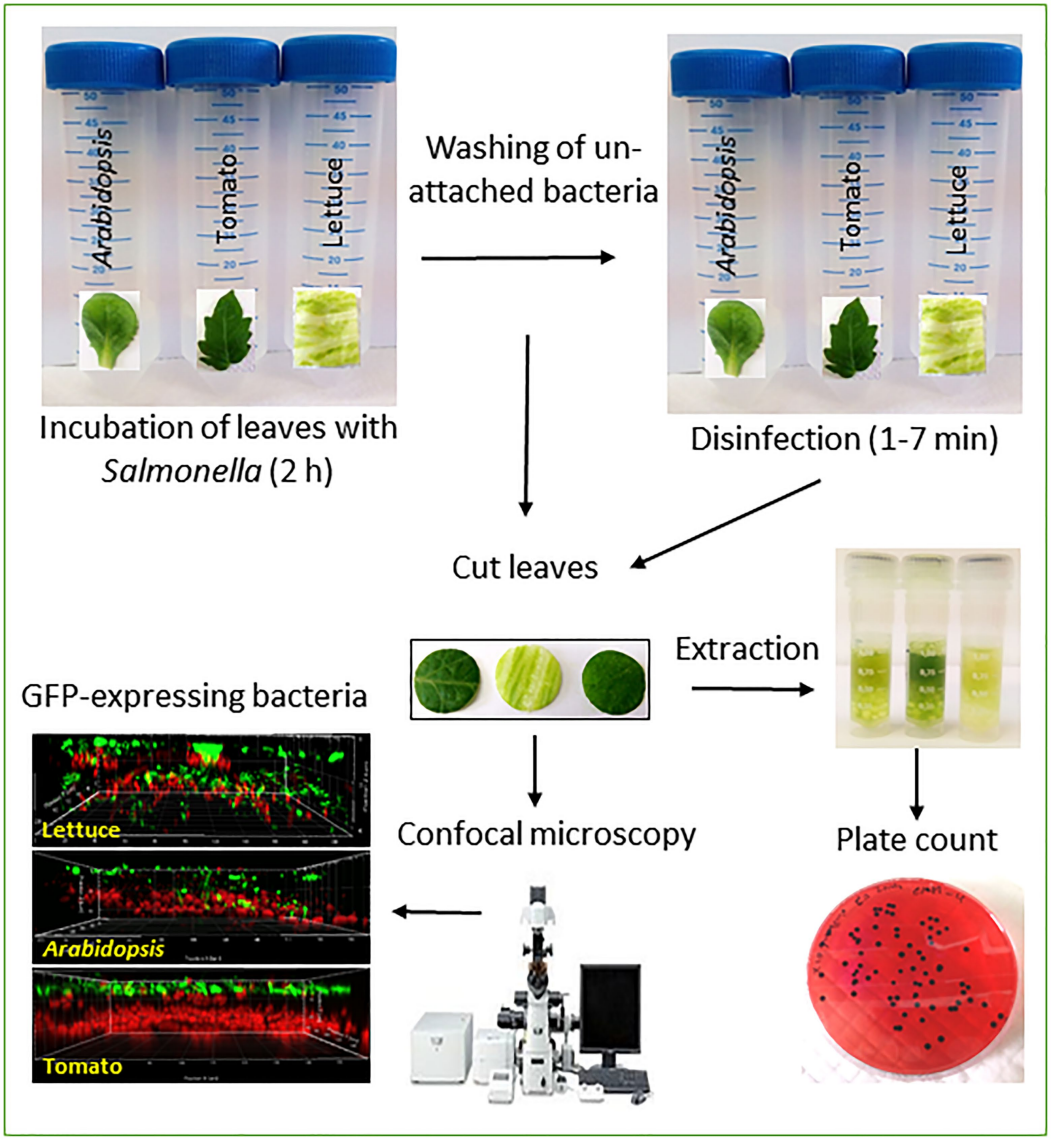
Outline of the methodologies used to assess leaf localization.

## Introduction

Foodborne illness is one of the most serious health problems worldwide, affecting public health and development (Newman et al., 2015). *Salmonella* spp. and pathogenic *Escherichia coli* strains are two of the main bacterial pathogens causing foodborne diseases (Takkinen et al., 2005; Friesema et al., 2008; Söderström et al., 2008; Gajraj et al., 2012). Worldwide, it is estimated that *Salmonella* is responsible for 80.3 million cases of foodborne illness (Majowicz et al., 2010). Raw fruits and vegetables are increasingly recognized as an important source of foodborne disease outbreaks in many parts of the world (Hanning et al., 2009; Wendel et al., 2009; Gajraj et al., 2012; Mritunjay and Kumar, 2015). Leafy vegetables were identified as the fresh produce commodity group of most significant concern from a microbiological safety perspective (Callejón et al., 2015; Mogren et al., 2018; Carstens et al., 2019; Kintz et al., 2019). Consequently, recent studies have focused on understanding the interactions between human pathogens and plants (Gu et al., 2011, 2013b; Barak and Schroeder, 2012; Cevallos-Cevallos et al., 2012; Schikora et al., 2012; Brandl and Sundin, 2013; Fletcher et al., 2013; Lim et al., 2014; Pollard et al., 2014; Holden et al., 2015; Fornefeld et al., 2017; Jacob and Melotto, 2019). Leaf attachment and internalization enable bacteria to get a foothold on the leaf surface and potentially reach the leaf interior (Barak and Schroeder, 2012; Deering et al., 2012; Erickson, 2012). The ability of both plant and human pathogens to reach the leaf interior is considered an important virulent trait, as internalized bacteria gain access to the nutrient-rich milieu within the leaf tissue and are protected against external environmental stresses, such as desiccation, irradiation, starvation, competition, and predation.

*Salmonella enterica* and *E. coli* can internalize the plants through natural openings, such as hydathodes, stomata, lenticels, lateral root emergence sites, or sites of biological or physical injury (Seo and Frank, 1999; Takeuchi and Frank, 2001; Solomon et al., 2002; Hora et al., 2005; Bernstein et al., 2007; Klerks et al., 2007; Underwood et al., 2007; Gomes et al., 2009; Kroupitski et al., 2009; Sharma et al., 2009; Barak and Schroeder, 2012; Deering et al., 2012; Gorbatsevich et al., 2013; Zheng et al., 2013; Gu et al., 2013a; Erickson et al., 2019; Riggio et al., 2019). A common method to assess leaf internalization is by taking advantage of the resistance of the internalized bacteria to surface disinfection (Deering et al., 2012; Erickson, 2012). Following inoculation of the pathogen of choice, surface-attached bacteria are killed by exposing the plants or plant’s organ to disinfecting agents. The plant tissue is then macerated to release the internalized bacteria and the disinfectant-protected bacteria are then enumerated by viable count, e.g., plating the homogenate on appropriate agar media. The viable count technique is straightforward and easy to perform and, consequently, it was widely adopted in studies assessing leaf internalization by enteric pathogens (For example, Duffy et al., 2005, Franz et al., 2007, Hadjok et al., 2008, Zhang et al., 2009, Erickson et al., 2010a,b, Gu et al., 2011, 2013a, Ge et al., 2013, Fakruddin et al., 2017). However, a major caveat of this method is that the results depend on the conditions used for surface sterilization, e.g., type of disinfectant(s) and treatment duration, which require validation for each specific combination of bacterial strain and plant cultivar. A literature review showed that only a few studies had validated the complete inactivation of surface-attached enteric bacteria, while in most cases, surface sterilization conditions were based on previously reported protocols, or the validation data were not presented (Table 1). Another approach to assess leaf internalization by foodborne pathogens is confocal microscopy. This method utilizes fluorescence-tagged bacteria and enables direct and precise localization of the bacteria within the leaf tissue. Nevertheless, it is time-consuming and requires expensive equipment (confocal microscope) and expertise. Commonly, confocal microscopy provides supportive data to confirm the internal localization of the tested bacteria and validate complete inactivation of surface-attached bacteria (Takeuchi and Frank, 2001; Duffy et al., 2005; Gu et al., 2011, 2013a; Erickson, 2012). In some cases, confocal microscopy may also provide quantitative data regarding leaf internalization (Kroupitski et al., 2009; Golberg et al., 2011).

**Table 1:** List of selected reports on leaf internalization of human enteric pathogens and the method used to study bacterial localization.

Plant	Pathogen	Disinfectant	References	Source of protocol	Confocal microscopy
Parsley	GFP-tagged *Salmonella* serovars Javiana, Rubislaw, and Anatum	2,000mg/liter sodium hypochlorite solution at 25°C for 3min	Duffy et al., 2005	Buchanan et al., 1999; used for *E. coli* O157:H7 internalization in apples	Yes
Lettuce	*Escherichia coli* O157:H7, *S*. Typhimurium strain MAE 110	−1% AgNO_3_ for 10s followed by two washing steps of 10S in water, −1% sodium hypochlorite for 5s followed by 5s in 70% EtOH and two washing steps	Franz et al., 2007	Franz et al., 2007	No
Lettuce	Five strains mixture of *E. coli* O157:H7 and 5 serovars of *Salmonella*	80% ethanol for 10s followed by immersion in 0.1% HgCl_2_ for 10min followed by five washing steps with water	Zhang et al., 2009	Zhang et al., 2009; using leaf prints	No
Lettuce, Spinach, and Parsely	*E. coli* O157:H7	−80% ethanol for 10s followed by immersion in 0.1% HgCl_2_ for 10min and washing with water, −1% AgNO_3_ for 10s followed by washing steps	Erickson et al., 2010a	Zhang et al., 2009; used for *E. coli* O157:H7 in lettuce (Franz et al., 2007); used for *E. coli* O157:H7 and S. Typhimurium in lettuce	No
Lettuce and Spinach	*E. coli* O157:H7	80% ethanol for 10s followed by 0.1% HgCl_2_ for 10min and washing steps, 1% AgNO_3_ for 10s followed by two washing steps	Erickson et al., 2010b	Zhang et al., 2009; used for *E. coli* O157:H7 in lettuce (Franz et al., 2007); used for *E. coli* O157:H7 and *S*. Typhimurium in lettuce	No
Lettuce Green onion	GFP-labeled *S*. Typhimurium	80% ethanol for 10s, 1% AgNO_3_ for 5min, washing with water	Ge et al., 2013	Franz et al., 2007; used for *E. coli* O157:H7 and *S*. Typhimurium in lettuce. Confirmed (data not shown)	No
Lettuce	*S*. Infantis	200ppm NaClO solution for 1min followed by washing steps	Zhang et al., 2016	FDA, 1998; Validated by comparing to the method of Zhang et al. (2009)	No
Tomato leaves	*S*. Montevideo	70% EtOH spray and allowed to dry under a flow hood until no visible solution remained	Miles et al., 2009	Not mentioned	no
Tomato plant	*S*. Typhimurium	70% alcohol for 20s and then 0.6% sodium hypochlorite for 10s followed by washing	Gu et al., 2011	Gu et al., 2011	Yes
Tomato leaves	*S*. Typhimurium SL1344 GFP-tagged	None	Golberg et al., 2011	Kroupitski et al., 2009	Yes
Tomato leaves	*S*. Typhimurium strain MAE110	70% alcohol for 15s following by water rinsing	Gu et al., 2013a	Validated by the authors	Yes
Tomato leaves	*S*. Newport	70% ethanol until runoff	Pollard et al., 2014	Not mentioned	No
Tomato leaves	*S*. Typhimurium strain MAE110	70% alcohol for 15s following by water rinsing	Gu et al., 2018	Not mentioned	Yes
Betel leaf	*S*. Enteritidis *S*. Typhimurium	80% ethanol for 10s, 1% AgNO_3_ for 5min, rinsing with water	Fakruddin et al., 2017	Franz et al., 2007; used for *E. coli* O157:H7 and *S*. Typhimurium in lettuce	No
Cucumber	Five *Salmonella* serovars	70% ethanol bath for 20min	Burris et al., 2020	Zheng et al., 2013; based on tomato leaf sterilization; validated in the lab; data not presented	No

In a previous study, employing confocal microscopy, we compared the internalization of *S. enterica* serovar Typhimurium, through stomata, in various leaves and found that it efficiently internalizes lettuce leaves but virtually failed to internalize tomato leaves, based on visualization of at least 360 microscopic leaf fields obtained from three plants (Golberg et al., 2011). It should be noted that numerous factors, such as bacterial strain, plant cultivar, growing conditions, age, epiphytic and endophytic flora, mode of inoculation, and other experimental factors, might affect the level and quantification of leaf internalization (Deering et al., 2012; Erickson, 2012; Gu et al., 2013b); yet validated data regarding the efficacy of a given protocol to assess bacterial internalization in different plant models are scarce. In the present study, we have employed an *in vitro* model system to systematically examine *Salmonella* stomatal internalization in tomato, lettuce, and *Arabidopsis thaliana* leaves using a specific *Salmonella* strain with three surface sterilization protocols, side by side with confocal microscopy validation. While all three plant species differ in their leaf structure and topography, the first two were shown to support significant levels of *Salmonella* internalization. In contrast, nearly no internalization was shown in tomato leaves by confocal laser microscopy (Golberg et al., 2011), making these leaves an ideal control system for assessing potential misinterpretation when using surface sterilization and viable count.

## Materials and Methods

### Bacterial Growth Conditions and Inoculum Preparation

Green-fluorescent protein (GFP)-labeled *S. enterica* serovar Typhimurium SL1334 strain (Kroupitski et al., 2009; Gu et al., 2013a) was used throughout the study. Bacterial culture was prepared and stored in Lysogeny broth (LB; Becton Dickinson, United States) supplemented with glycerol at −70°C, as described (Kroupitski et al., 2009). For each experiment, fresh culture was prepared by plating the bacteria on a new LB plate supplemented with 100mg/ml streptomycin and 10mg/ml gentamicin for 24h at 37°C. Two to three single colonies were as inoculated into LB broth devoid of NaCl (LBNS) and grown at 37°C with shaking (150rpm) for 18–20h. Cultures were washed twice with sterile saline (0.85% NaCl) by centrifugation at 2,700g for 10min, and the final pellet was resuspended in sterile saline. Bacterial concentration was determined by plating × 10-fold serial dilutions on LB agar supplemented with the two antibiotics.

### Preparation of Leaves

*A. thaliana* (Col-0) plants were grown in a potting mix containing (w/w) 70% peat, 30% perlite, supplemented with slow-release fertilizer (7,611, Even-Ari, Israel) under 10-h light / 14-h dark (short day) photoperiod, at 22°C with a relative humidity of 55–60% and light intensity of 130μmolm^−2^ S^−1^. Tomato plants (*Solanum Lycopersicon*), cultivar M82, were grown in Green quality soil mix, Tuff soil (Merom Golan, Israel) under 16-h light / 8-h dark, at 25°C. *Arabidopsis* and tomato leaves of 4- and 6-weeks old plants, respectively, were aseptically cut from the plants, and whole leaves or leaflets were used for the experiments. Fresh iceberg lettuce (*Lactuca sativa*) was obtained from a local retail store and used on the day of purchase or stored in the refrigerator for up to 12h before use. The outermost leaves of the lettuce head were aseptically removed, and two or three inner leaves were taken for the experiments. The lettuce leaves were cut into *ca*. 3- by 3-cm pieces using a sterile scalpel, as described before (Kroupitski et al., 2009), and individual pieces were used for the experiments.

### Inoculation of Leaves

Inoculation of leaves was performed, essentially as described before (Kroupitski et al., 2009, 2011, 2019; Golberg et al., 2011), except for the incubation temperature. Briefly, a single tomato leaflet, *Arabidopsis* leaf, or lettuce piece were each submerged in a single 50-ml sterile polypropylene tube (Labcon, Petaluma, CA) containing 30-ml saline. The leaves were illuminated for 20min under a light intensity of 150-μE m^−2^ s^−1^ at room temperature, and then, the saline was removed and replaced with a bacterial suspension containing *ca*. 10^8^ *Salmonella* CFU/ml saline. While this high inoculum does not represent real-life conditions, such high inocula were previously used to study *Salmonella* internalization *in vivo* (Gu et al., 2011, 2013a) and *in vitro* (Kroupitski et al., 2009, 2011, 2019; Golberg et al., 2011). The incubation proceeded for 2h at 40°C, a temperature that increases stomatal openings in multiple species (Kostaki et al., 2020) to facilitate *Salmonella* internalization. The leaf samples were washed twice by dipping in fresh sterile saline for 1min each time to remove unattached bacteria. *Salmonella* attachment to the leaf surface and internalization was analyzed by confocal microscopy and viable count, as described below. Each experiment included three leaves (repeats) of the same plant, each in a single tube and the three plants species were processed on the same day. The experiments were repeated twice for all plants on different days.

### Determination of *Salmonella* Internalization Using Surface Disinfection

Surface disinfection was performed using one of the three disinfectants, 1% sodium hypochlorite (Bio-Lab Ltd., Jerusalem, Israel), 1% silver nitrate (Bio Basic Ltd. Toronto, Canada), and 70% ethanol (Gadot-Group, Netanya, Israel). Briefly, whole leaves of *Arabidopsis* and tomato or lettuce leaf samples were submerged in 20-ml disinfectant solution with gentle agitation for 7min. Leaf samples were taken out after 1, 3, 5, and 7min and washed extensively by dipping the leaves four times (1min each) in 20-ml sterile double-distilled water (SDDW) to remove the residual disinfectant solution. In order to avoid interference by bacteria that may enter through the cut tissues, an internal leaf disks (2-cm^2^ area) were excised from the three leaves (*Arabidopsis*, tomato, and iceberg lettuce) using a sterile cork-borer. The leaf disks were aseptically cut into two identical pieces with a sterile scalpel, one was taken for bacterial extraction and viable count, and the other was taken for confocal microscopy. A high-speed benchtop homogenizer Fast Prep^®^-24 (MP-Biomedicals, Solon Ohio, United States) was used for the homogenization of the leaf samples in 2-ml micro-tubes (MP-Biomedicals, Solon Ohio, United States) containing glass beads and 500μl of buffer peptone water (BPW; Becton Dickinson, France, United States). Homogenization conditions were 4,000rpm for 40s at room temperature. Homogenate samples (100μl) and 10× serial dilutions were spread plated into Xylose-Lysine-Desoxycolate (XLD; Becton Dickinson, France, United States) agar supplemented with streptomycin and gentamicin in order to enumerate internalized *Salmonella* cells that presumably survived the disinfection treatment. Inoculated leaves suspended for up to 7min in SDDW without disinfection and then washed in fresh SDDW served as non-treated control to determine the initial number of leaf-associated bacteria. *Salmonella* counts of control and treated samples were converted to log CFU/cm^2^.

### Determination of *Salmonella* Internalization Using Confocal Microscopy

Fluorescently-labeled *Salmonella* cells were visualized using a confocal laser-scanning microscope (Olympus IX81; Olympus, Tokyo, Japan) with a 40X objective lens and a numerical aperture of 0.7. *Salmonella* localization of fluorescent bacteria on the leaf surface and in internal leaf tissues was determined in 30 randomly selected microscopic fields per leaf, as described before (Kroupitski et al., 2009). Briefly, quantification of the surface-attached and internalized bacteria was done by calculating the percentage of microscopic fields that harbor ≥1 internal or surface-attached *Salmonella* cells in 30 fields and is presented as the incidence (%) of *Salmonella* on the surface and internal tissues, as described previously (Kroupitski et al., 2009). The mean incidence of *Salmonella* was calculated based on two independent experiments, each containing three technical repeats.

### Statistical Methods

All experiments were performed in triplicates (three different leaf samples) and repeated two times on different days. Statistical analysis was performed using the JMP software package version 14 (SAS Institute Inc., Cary, NC, United States). Incidence data were arcsine-transformed before analysis and residual data for logarithm of CFU/area after analysis were examined to determine normality and equality of variances. Two-way ANOVA was used to analyze the effect of disinfectant, time, and their interaction. After significant interaction was discovered, pairs of disinfectant-time means were compared by the Tukey-Kramer test (alpha=0.05).

## Results

### Determination of Leaf Internalization Using Surface Sterilization and Viable Count in Various Leaves

**Figure 1:**
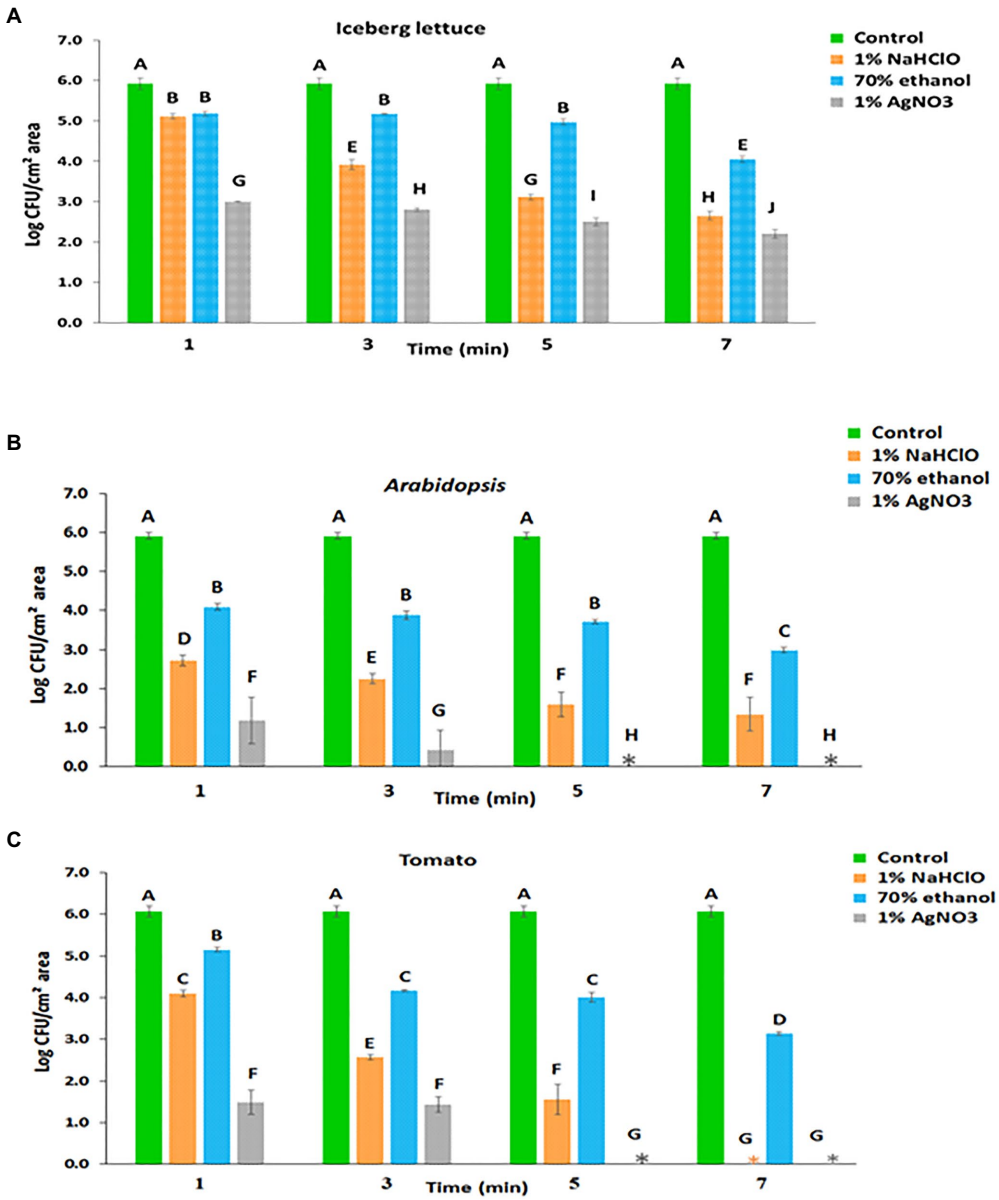
Leaf internalization based on viable count following surface disinfection of **(A)** lettuce, **(B)** *Arabidopsis*, and **(C)** tomato. Mean and standard deviation values of two individual experiments each with three technical repeats are presented. Different letters indicate significant differences (*p*<0.05) in control and treated leaf samples, for a given plant, according to the Tukey-Kramer multiple comparison test.

Leaf internalization was initially studied in lettuce and *Arabidopsis*, which were previously shown to support a high level of *Salmonella* internalization (Kroupitski et al., 2009; Golberg et al., 2011). Incubation of lettuce leaves with *S. enterica* serovar Typhimurium for 2h resulted in a surface colonization density of 5.92±0.15 log CFU/cm^2^ (Figure 1A), representing both surface-attached and leaf-internalized bacteria. Treatment of the inoculated lettuce leaf with 1% NaHClO for 1, 3, 5, and 7min reduced the number of viable *Salmonella* cells from 5.92±0.15 log CFU/cm^2^ to 5.11±0.07, 3.92±0.1, 3.11±0.08, and 2.65±0.11 log CFU/cm^2^, respectively (Figure 1A).

After 70% ethanol treatment for 1, 3, 5, and 7min, surviving *Salmonella* counts were reduced from 5.92±0.15 log CFU/cm^2^ to 5.18±0.06, 5.17±0.01, 4.97±0.07, and 4.05±0.08 log CFU/cm^2^, respectively (Figure 1B). Unlike ethanol, surface disinfection with 1% AgNO_3_ resulted in a higher killing rate. Treatment with 1% AgNO_3_ for 1, 3, 5, and 7min reduced the number of viable *Salmonella* cells from 5.92±0.15 log CFU/cm^2^ to 3.00±0.01, 2.80±0.04, 2.50±0.10, and 2.20±0.11 log CFU/cm^2^, respectively (Figure 1A).

Incubation of *Arabidopsis* leaves with *Salmonella* for 2h resulted in a surface colonization density of 5.91±0.09 log CFU/cm^2^ of leaf-associated bacteria (Figure 1B). Following surface disinfection with 1% NaHClO for 1, 3, 5, and 7min, the counts were reduced to 2.72±0.14, 2.25±0.13, 1.60±0.32, and 1.34±0.43 log CFU/cm^2^, respectively (Figure 1B). Surface disinfection with 70% ethanol for 1, 3, 5, and 7min reduced the numbers of viable *Salmonella* cells to 4.09±0.08, 3.88±0.11, 3.72±0.06, and 2.98±0.07 log CFU/cm^2^, respectively (Figure 1B). Disinfection with 1% AgNO_3_ resulted in a higher inactivation of leaf-associated *Salmonella*, and after treatment for 1 and 3min, the counts were reduced to 1.18±0.60 and 0.43±0.51 log CFU/cm^2^, respectively. Longer incubation times resulted in the inactivation of all leaf-associated *Salmonella* cells (Figure 1B).

Incubation of tomato leaves with *Salmonella* suspension for 2h resulted in a surface colonization density of 6.06±0.14 log CFU/cm^2^, representing the total number of leaf-associated *Salmonella* (Figure 1C). This value corresponds to the sum of surface-attached and internalized bacteria. The number of internalized *Salmonella* was assessed by the viable count technique following leaf disinfection. Treatment with 1% NaHClO for 1, 3, and 5min duration resulted in the survival of 4.10±0.08, 2.57±0.06, and 1.56±0.37 log CFU/cm^2^ leaf area, respectively (Figure 1C), which presumably represent internalized bacteria. Treatment duration of 7min resulted in complete *Salmonella* eradication. Treatment with 70% ethanol for 1, 3, 5, and 7min resulted in the survival of 5.15±0.05, 4.17±0.03, 4.01±0.12, and 3.14±0.04 log CFU/cm^2^, respectively (Figure 1C). Finally, the treatment of inoculated tomato leaves with 1% AgNO_3_ for 1 and 3min resulted in 1.48±0.29 and 1.43±0.19 log CFU/cm^2^, respectively; while longer incubation times of 5 and 7min resulted in complete *Salmonella* inactivation (Figure 1C).

### Determination of *Salmonella* Internalization by Confocal Microscopy

In parallel to the bacteriological studies, *Salmonella* internalization was studied by confocal microscopy using the other part of the same leaf piece used for assessing internalization by the viable count technique. Both non-treated and surface-disinfected leaf samples were utilized in these studies.

**Figure 2:**
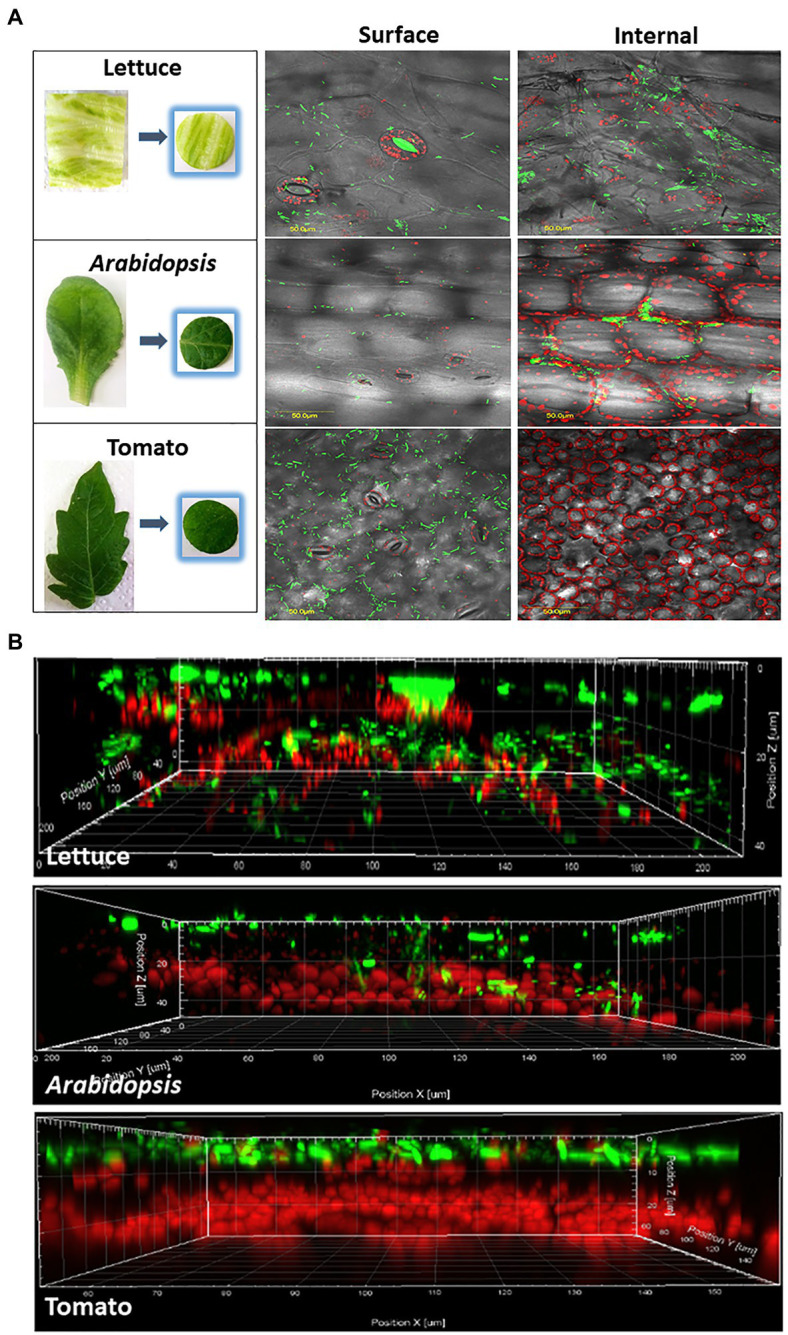
Confocal microscopy visualization illustrating epiphytic and endophytic localization of GFP-labeled *Salmonella* cells in representative leaves of iceberg lettuce, *Arabidopsis*, and tomato. Panel **A** shows images taken from the surface of the leaves, and a stack of fluorescent images along a *z*-section taken every 1.2μm to a depth of 100μm below the surface. All images were overlaid with differential interference contrast (DIC) images taken from the same location in each leaf. Bar denotes 50μm. Panel **B** shows a z-section model of the same leaves, demonstrating the location of bacteria (green) on and within the leaf tissues. Red fluorescence indicated autofluorescence of the chloroplasts.

Confocal microscopy studies were performed with lettuce, *Arabidopsis*, and tomato leaves (Figure 2 and Table 2). In lettuce leaves, *Salmonella* cells showed comparable distribution between the leaf surface and the leaf interior. The incidence of *Salmonella* cells on the leaf surface was 100%, while the incidence of *Salmonella* cells underneath the leaf surface was 92±1% (Table 2). Treatment of the leaves with each of the three disinfectants for 1 or 7min resulted in most cases in a substantial reduction in the incidence of fluorescent cells, both on the leaf surface and within the leaf interior. Ethanol treatment for 1min reduced the incidence of fluorescent cells on the leaf surface to 58±3%, yet it did not affect the incidence of endophytic *Salmonella*. The two other disinfectants reduced the incidence of fluorescent *Salmonella* both on the leaf surface as well as in the leaf interior during longer exposure times.

In the case of *Arabidopsis*, confocal microscopy of leaves incubated with *Salmonella* showed an incidence of *Salmonella* of 100% on the leaf surface and 42±6% underneath the surface (Figure 2 and Table 2). Surface disinfection with 1% NaHClO, 70% ethanol, and 1% AgNO_3_ for 1min resulted in a decrease in the incidence of surfaced-attached *Salmonella* from 100% to 41±3.6, 55±3.5 and 57±7%, respectively, and a further reduction of surface-attached bacteria occurred after a longer exposure time (Table 2). However, fluorescent *Salmonella* cells were still observed on the leaf surface. Treatment of the leaves with 70% ethanol for 7min reduced the incidence of endophytic *Salmonella* from 42±6% to 31±5.7%, while treatment with 1% NaHClO for 7min and 1% AgNO_3_ for 5min resulted in complete loss of fluorescence, inferring *Salmonella* inactivation.

In contrast to the findings with lettuce and *Arabidopsis* leaves, imaging of tomato leaves following incubation with fluorescent *Salmonella* revealed no endophytic colonization. All leaf-associated *Salmonella* cells were confined to the leaf surface (Table 2 and Figure 2). Disinfection with 1% NaHClO for 1min resulted in reducing the incidence of surface-associated *Salmonella* from 100 to 35±1.5%, while 7min exposure resulted in the loss of fluorescence, inferring a complete inactivation of the pathogen (Table 2). Exposure of the leaf to 1% AgNO_3_ for 1 and 5min resulted in similar effects, while ethanol (70%) had a milder effect. It reduced the incidence of fluorescent *Salmonella* to 55±1% after 1min and to 11±2% after 7min.

## Discussion

Human pathogens can colonize plants and persist on and sometimes within various plant’s tissues, and upon consumption may cause foodborne diseases (Barak and Schroeder, 2012; Deering et al., 2012; Erickson, 2012; Brandl and Sundin, 2013; Holden et al., 2015; Fornefeld et al., 2017; Jacob and Melotto, 2019; Roy and Melotto, 2019; Schierstaedt et al., 2019). Accordingly, accurate determination of the localization of human pathogen on or within leaves is vital for basic science as well as for developing new strategies for preventing and intervening to address the problem of fresh produce contamination.

The determination of bacterial internalization in a plant is a function of, among others, the method used to assess bacterial localization (Deering et al., 2012; Erickson, 2012). Ultimately, surface sterilization should completely inactivate external bacteria while leaving internalized bacteria intact. Still, only a few studies have systematically validated the efficacy of surface sterilization to kill surface-attached bacteria. In one such study, 13 disinfection conditions/methods were compared for their effectiveness in killing GFP-tagged *E. coli* O157:H7 on lettuce leaf surfaces using leaf imprints on agar media. Dipping in 80% ethanol for 10s followed by immersion in 0.1% HgCl_2_ for 10min was reported to be the most effective disinfection method for inactivating both *E. coli* and *Salmonella* strains (Zhang et al., 2009). However, no confocal microscopy study corroborated the results. Many studies have adopted previously reported protocols to inactivate surface-attached bacteria, even when utilizing different plants and/or bacterial strains (see Table 1). Bacteria may vary in their intrinsic tolerance to disinfectants (Morente et al., 2013) and may preferentially reside at unique leaf-specific microsites (Beattie and Lindow, 1995; Erickson, 2012), which may facilitate the protection of the colonized bacteria against disinfection (Andrews and Harris, 2000; Erickson et al., 2010a; Erickson, 2012). Consequently, a disinfection protocol developed for inactivating a specific *Salmonella* strain on the leaves of a particular plant cultivar may not fit all. Evidently, when a partial inactivation is achieved, some surface-residing bacteria may be misclassified as internal bacteria, while truly internalized bacteria killed due to permeation of the disinfectant into the intact leaf tissue may be mistakenly regarded as surface-attached bacteria.

The present study provides data from a systematic comparison of leaf internalization through stomata by a GFP-tagged *Salmonella* Typhimurium strain in the leaves of the three plant species using surface sterilization and plate-count technique. The study did not compare leaf internalization among plants but rather the effect of the various disinfection protocols on leaf internalization in each plant species. We used three disinfectants (1% NaHClO, 1% AgNO_3_, and 70% ethanol), commonly applied, alone or in combination with others, for sterilizing plant surfaces (Franz et al., 2007; Erickson et al., 2010b; Gu et al., 2011, 2013a; Erickson, 2012; Ge et al., 2013; Fakruddin et al., 2017). To simplify the comparison between the protocols, we used a single concentration of the disinfectants, each time, and compared the effect of the sterilization time (1 to 7min) on quantifying viable bacteria, apparently representing internalized *Salmonella* cells. In parallel to the viable counts measurements, we utilized confocal microscopy to accurately assess bacterial localization on or within the leaf tissue.

We initially examined leaf internalization in iceberg lettuce leaves previously shown by confocal microscopy to support *Salmonella* internalization (Kroupitski et al., 2009; Golberg et al., 2011). Indeed, confocal microscopy confirmed a high incidence of internalization (92%) in non-disinfected leaves; however, surface disinfection with all three agents resulted in reducing fluorescence, suggesting that the disinfectants seemingly penetrated the leaf tissues to some degree and injured the cells (Table 2). A substantial decrease in the number of apparent internalized bacteria was observed using the plate-count method following 1 to 7min treatment (Figure 1). The determination of leaf internalization by surface disinfection and viable count showed wide variations in the number of apparently internalized bacteria in leaves of each plant species, depending upon the type of the disinfectant and the treatment duration. These differences are likely attributed to the increased killing of leaf-associated bacteria with time or to the transition of a portion of the *Salmonella* population to the viable but non-culturable (VBNC) state (Zhao et al., 2017). The observation of fluorescent cells on the leaf surface does not provide a clear indication regarding the presence of disinfection-tolerant bacteria, since the *Salmonella* strain carried a stable GFP (Kroupitski et al., 2009), which may continue to emit fluorescence in VBNC bacteria, as well as in dead cells with intact GFP. Further studies using methods that can discriminate between live and dead bacteria are needed to determine the physiological status of the treated fluorescent bacteria on the leaves’ surface. Still, the possible entry of *Salmonella* into a VBNC state in the plant environment (Winfield and Groisman, 2003) may lead to underestimation of both attachment and internalization when using the viable count assay alone.

**Table 2:** Incidence of fluorescent *Salmonella* cells in various leaf localization using confocal microscopy.

		Incidence of *Salmonella* in leaf localization (%)			
		Surface		Internal	
	Treatment/Time (min)	1	7	1	7
Iceberg lettuce	Control (water)	100^A^	100^A^	92±1^a^	92±1^a^
	1% NaHClO	50±1.5^D^	16±2.5^E^	63±5^c^	41±2.5^d^
	70% ethanol	58±3^B^	16±4^E^	91±1.5^a^	77±2.6^b^
	1% AgNO_3_	59±4^B^	54±1^C^	67±5^bc^	66±5^bc^
	
*Arabidopsis*	Control (water)	100^A^	100^A^	42±6^a^	42±6^a^
	1% NaHClO	41±3.6^C^	12±3.2^D^	16±3.5^c^	0^d^
	70% ethanol	55±3.5^BC^	14±2.6^D^	42±2.6^a^	31±5.7^b^
	1% AgNO_3_	57±7^B^	15±4.7^D^	32±5^b^	0^d^
	
Tomato	Control (water)	100^A^	100^A^	0^a^	0^a^
	1% NaHClO	35±1.5^C^	0^E^	0^a^	0^a^
	70% ethanol	55±1^B^	11±2^D^	0^a^	0^a^
	1% AgNO_3_	38±7.6^BC^	0^E^	0^a^	0^a^

a *For each plant, means without a common uppercase letter or without a common lowercase letter differ significantly by the Tukey-Kramer multiple comparison test (p<0.05) with regard to the incidence of Salmonella of surface-attached and internal Salmonella, respectively*.

Based on the confocal microscopy studies, *Salmonella* displays a lower incidence of leaf internalization in *Arabidopsis* than in lettuce (Table 2). Likewise, the viable count method demonstrated lower numbers of viable bacteria during all treatment times (Figure 1B,C). All three agents displayed comparable surface disinfection effectiveness; however, they varied significantly in the apparent internalization (Table 2). A 7-min treatment with 1% NaHClO or 1% AgNO_3_ resulted in the complete loss of fluorescent cells inside the leaf, suggesting that they efficiently penetrated the leaves and injured the internalized bacteria.

In a previous report, we were not able to show internalization of the same *Salmonella* strain in tomato leaves (Golberg et al., 2011). Consequently, the assessment of tomato leaf internalization, side by side, by the two methodologies provided a unique opportunity to assess the suitability of the tested disinfection conditions inactivate bacteria in the leaf surface. Evaluation of *Salmonella* internalization by confocal microscopy, with no surface sterilization, confirmed our inability to demonstrate the internalization of *Salmonella* in these tomato leaves with the techniques used. Usage of 1% NaHClO for 1 to 7min resulted in different numbers of apparent internalized bacteria, ranging from 4 logs CFU/cm^2^ to 0, respectively. Parallel confocal microscopy analysis of the treated leaf samples confirmed the lack of detection of leaf internalization, suggesting that only 7-min treatment resulted in sufficient killing of external bacteria in this model system. The use of 70% ethanol as a sole disinfectant for up to 7min failed to inactivate all external bacteria, as determined by viable counts, thus mistakenly suggesting the internalization of about 3 log CFU/cm^2^. Treatment with 1% AgNO_3_ resulted in substantial inactivation of surface-attached bacteria in 1 and 3min treatment, while treatment duration of 5 and 7min was sufficient to kill all external bacteria, hence providing results comparable to those obtained by confocal microscopy. These findings indicate that non-validated surface sterilization conditions may lead to misinterpretation of the actual number of internalized bacterial cells. Notably, the apparent lack of leaf internalization of the tested *S. typhimurium* strain (SL 1344) in the tomato cultivar used in this study (*Solanum lycopersicon* cv. M82), as well as in *S. lycopersicon* cv. MP1, tested previously (Golberg et al., 2011), calls for further research. It is particularly interesting to examine whether the two cultivars are naturally resistant to leaf internalization of other *Salmonella* serovars and strains under more natural tomato growing conditions. Elucidation of the mechanisms involved in the inhibition of leaf internalization might prove important for understanding human pathogen-plant interactions and developing new mitigation strategies for *Salmonella* internalization.

Surface disinfection by treatment with 1% AgNO_3_ was less effective in lettuce compared to tomato leaves. These differences are likely correlate to specific leaf features, such as surface morphology and/or physico-chemical properties known to impact leaf colonization (Beattie and Lindow, 1995; Andrews and Harris, 2000; Beuchat, 2002; Yadav et al., 2005; Heaton and Jones, 2008; Leveau, 2009; Cevallos-Cevallos et al., 2012). Previous studies have already noted that the attachment of bacteria to specific microenvironments on the leaf, such as cavities and crevices on the leaf surface, may favor the persistence of surface-attached bacteria following disinfection (Gomes et al., 2009; Deering et al., 2012; Erickson, 2012).

Altogether, this is the first time a systematic study reported a comparison of three surface sterilization protocols in leaves of three plants, side by side, with a confocal microscopy study. While the selection of an optimal disinfection protocol for each of the three plants was beyond the scope of this study, we have demonstrated the dependency of the apparent bacterial internalization on the disinfection conditions and shown the impact of the quantification method on the extent of leaf internalization.

It should be noted that entry of bacterial pathogens into the leaf tissue might occur through stomata, hydathodes, and injured tissues or by transport through the roots and stem (Erickson, 2012; Gu et al., 2013a; Melotto et al., 2017). In the present study, we utilized specific *in vitro* inoculation and experimental conditions to compare the effect of three surface disinfection protocols on *Salmonella* internalization through stomata. The study was not designed to investigate other factors that might affect bacterial internalization nor the different mode of leaf internalization. Therefore, we suggest interpretation of our results with caution, especially when comparing to other studies that used different inoculation models and surface disinfection protocols.

Whole leaves or leaflets were used for inoculation in the case of *Arabidopsis* and tomato, respectively; however, in the case of lettuce, square leaf pieces were used, which potentially may enable direct access of bacteria into the apoplast through the injured tissue. However, previous confocal microscopy observations showed a limited penetration of *Salmonella* through the cut tissues (data not shown), which did not affect the internal leaf tissue used for bacterial enumeration.

While the use of confocal microscopy to determine bacterial localization is critical for confirming leaf internalization, this technique is limited to high concentrations of fluorescent cells, which may not represent natural contamination scenarios. Furthermore, unlike the bacteriological technique, quantification of internalization by confocal microscopy relies on a limited number of microscopic fields, which might bias the results. On the other hand, the viable count technique, but not confocal microscopy, may be prone to changes in the physiological status of the leaf-associated bacteria, such as transition into the VBNC state.

Conclusion

In conclusion, the data of the internalization model presented here emphasize the need for a careful examination and calibration of the surface sterilization protocol, including testing of different disinfectant’s concentrations as well as combinations of disinfectant, particularly when a new plant system and bacterial strain are studied, where the sterilization conditions may need to be adjusted prior to further experimentation. Our findings may also be relevant to studies aimed at the isolation and characterization of endophytic microorganisms, which utilize an initial surface sterilization step to inactivate external plant microorganisms.

## Data Availability Statement

The original contributions presented in the study are included in the article/supplementary material, and further inquiries can be directed to the corresponding author.

## Author Contributions

MC conducted the experiments, performed the data analysis, and drafted the manuscript. YK and RG assisted with the experiments and contributed to data analysis. EB performed the confocal microscopy studies. MM contributed to the discussion and reviewed the manuscript. SS-S conceived the study and wrote the manuscript. All authors read and approved the manuscript.

## Funding

The study was partially supported by the NIFA-BARD Collaborative Research Program awarded to MM and SS-S. The research of SS-S was supported by the United States – Israel Binational Agricultural Research and Development Fund, NB-8316-16, and the research of MM was supported by the National Institute of Food and Agriculture – USDA Fund no. 2017–67017-26180.

## Conflict of Interest

The authors declare that the research was conducted in the absence of any commercial or financial relationships that could be construed as a potential conflict of interest.

## Publisher’s Note

All claims expressed in this article are solely those of the authors and do not necessarily represent those of their affiliated organizations, or those of the publisher, the editors and the reviewers. Any product that may be evaluated in this article, or claim that may be made by its manufacturer, is not guaranteed or endorsed by the publisher.
